# Mobile Bearing Total Knee Arthroplasty for Valgus Knee Osteoarthritis with Permanent Patellar Dislocation: A Case Report and Review of the Literature

**DOI:** 10.1155/2017/1230412

**Published:** 2017-03-29

**Authors:** Kohei Kamada, Tomoyuki Matsumoto, Koji Takayama, Daisuke Araki, Shingo Hashimoto, Shinya Hayashi, Takehiko Matsushita, Ryosuke Kuroda

**Affiliations:** Department of Orthopaedic Surgery, Kobe University Graduate School of Medicine, 7-5-1, Kusunoki-cho, Chuo-ku, Kobe, Hyogo 650-0017, Japan

## Abstract

Permanent patellar dislocation with tibiofemoral joint osteoarthritis is a relatively rare condition. To treat this condition, total knee arthroplasty with proximal or distal realignment of the extensor mechanism has been reported. We report a challenging case of an 80-year-old woman diagnosed with permanent patellar dislocation with tibiofemoral joint osteoarthritis treated by a mobile bearing total knee arthroplasty utilizing navigation system. Lateral retinaculum release was performed to improve patellar tracking; other proximal or distal realignment of the extensor mechanism was not necessary. Postoperative radiographs show stable patellar tracking and recurrent patellar dislocation was not observed. This clinical case indicates that the implant's precise alignment and rotation during total knee arthroplasty could settle anatomical abnormalities of permanent patellar dislocation and the mobile bearing insert could contribute to stabilizing patellar tracking.

## 1. Introduction

Permanent patellar dislocation with tibiofemoral (TF) joint osteoarthritis (OA) is a relatively rare condition. To treat this condition, total knee arthroplasty (TKA) with proximal or distal realignment of the extensor mechanism has been reported [1–5]. To our knowledge, however, there is no report about TF joint OA with permanent patellar dislocation treated by mobile bearing TKA with lateral retinaculum release. Mobile bearing TKA can theoretically adjust rotational malalignment by its self-align feature and improve patellar tracking and patellofemoral contact stress [6]. Our case demonstrated the therapeutic potential of mobile bearing TKA with lateral retinaculum release for TF joint OA with permanent patellar dislocation.

## 2. Case History

An 80-year-old woman who was diagnosed with permanent patellar dislocation after a fall ten years before was referred to our hospital with a chief complaint of severe left knee pain. Her left knee showed permanent dislocation of the patella and severe valgus deformity. Tenderness was present in the lateral joint space. Passive range of motion in the left knee was from 20° to 75° with no extension lag. The patella remained dislocated throughout the full range of movement and could not be reduced manually. Knee Society Knee Score (KSKS) was 73 points, and Knee Society Function Score (KSFS) was 54 points. There was no significant medical history. Regarding family history, her younger sister had OA of the patellofemoral joint.

**Figure 1:**
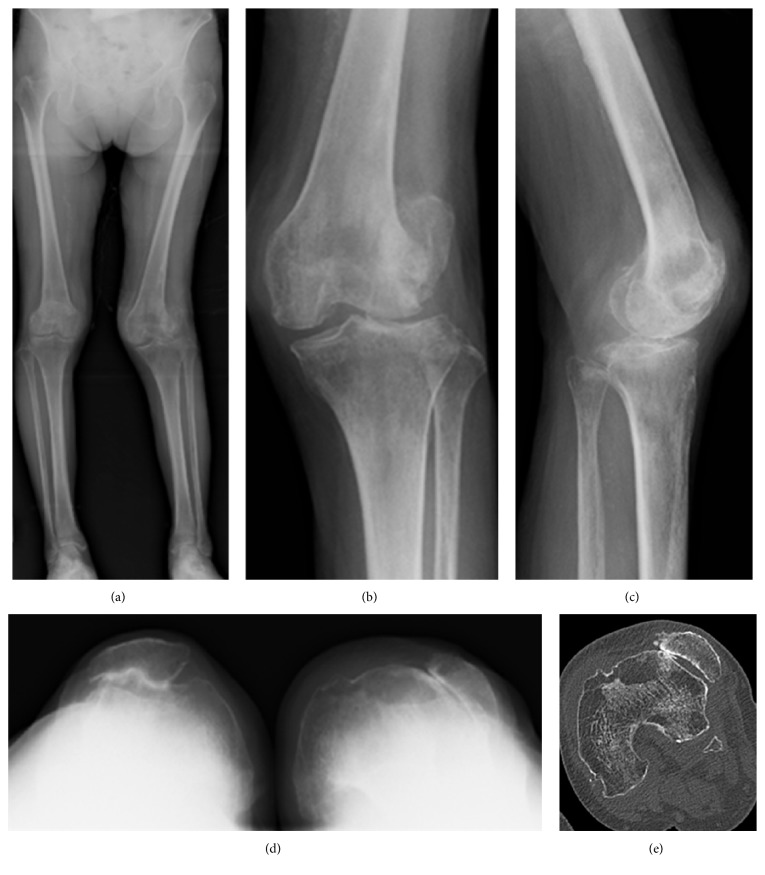
Preoperative plain radiograph and computed tomography. Anteroposterior long leg standing image of both legs (a), anteroposterior one leg standing image of the left knee (b), lateral one leg standing image of left knee (c), skyline view of patella at 30 degrees of knee flexion (d), and preoperative computed tomography of the left knee (e).

Long leg standing radiographs showed severe lateral OA, a femorotibial angle (FTA) of 160°, and a hip-knee-ankle angle (HKA) of valgus 12°. Skyline view radiographs showed the patella was dislocated to the lateral side of the lateral femoral condyle, the central ridge was absent, and the trochlear groove was hypoplastic (sulcus angle was 169°) (Figures 1(a)–1(d)). Computed tomography (CT) scan showed that the tibial tubercle-trochlear groove distance was 22 mm, and the surgical epicondylar axis (SEA) was rotated 7.6° externally from the posterior condylar axis (PCA) (Figure 1(e)).

**Figure 2:**
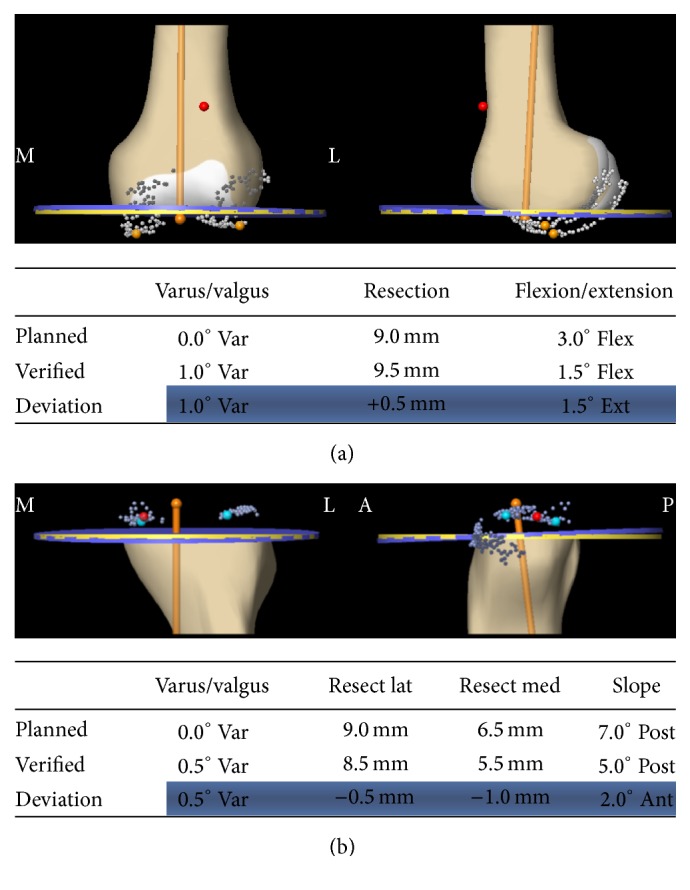
The data of navigation system. The screenshot of navigation system in the distal femur (a) and the proximal tibia (b).

Cruciate retaining (CR) mobile bearing TKA (ATTUNE™ TKA system; DePuy, Warsaw, IN, USA) was performed under general anesthesia. The medial parapatellar approach was used. The distal femur and proximal tibia were cut vertically to the mechanical axis using navigation system (VectorVision CT-free knee; BrainLAB, Munich, Germany). The distal femur was cut 0.5 degrees in varus and 1.5 degrees in flexion and the proximal tibia was cut 0.5 degrees in varus and 5.0 degrees in posterior slope when we confirmed the precision of osteotomy with navigation system (Figures 2(a) and 2(b)). It was approximately as planned. The femoral component was externally rotated seven degrees to the PCA and parallel to the SEA. The tibial tray was placed parallel to Akagi's line [7]. The iliotibial tract was released at the level of the femorotibial joint to correct the valgus deformity. The patellar implant was placed on the medial side of the patella and lateral retinaculum release was performed from outside the vastus lateralis to the distal patella. The medial soft tissue procedures were not performed. The joint capsule and skin were closed after confirming stable patellar tracking throughout the full range of motion.

**Figure 3:**
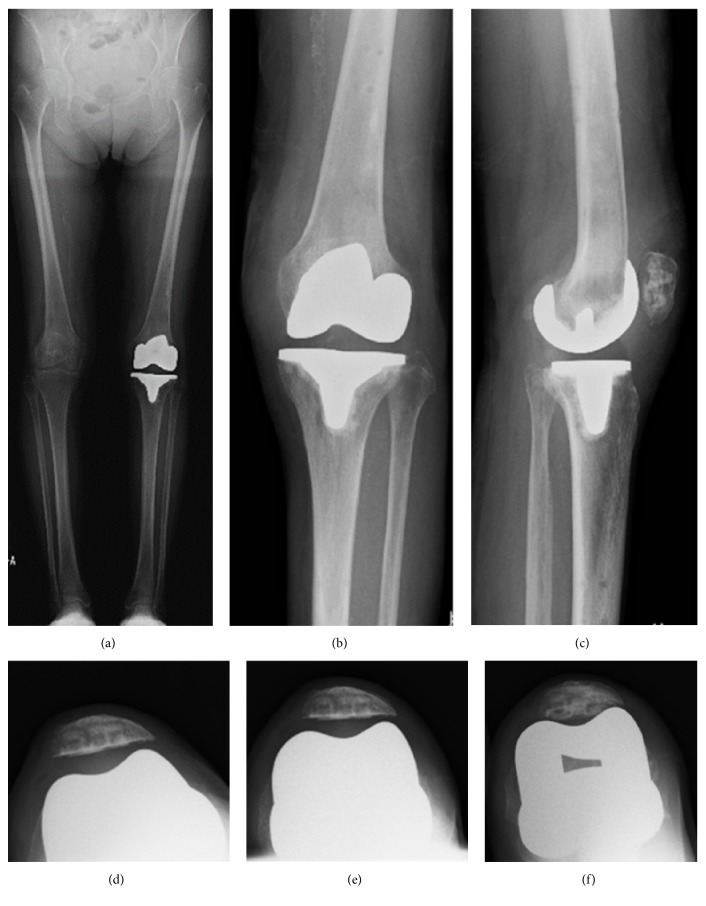
Postoperative plain radiograph. Anteroposterior long leg standing image of both legs (a), anteroposterior one leg standing image of left knee (b), lateral one leg standing image of left knee (c), skyline view of patella at 30 degrees of knee flexion (d), 60 degrees of knee flexion (e), and 90 degrees of knee flexion (f).

As in typical TKA cases, weight bearing and range of motion exercise was allowed as tolerated the day after surgery. One year postoperatively, the patient had a range of motion of 5° to 110° and no extension lag. KSKS and KSFS improved to 93 points and 95 points, respectively. She can walk smoothly without a cane and patellar dislocation did not recur. Postoperative plain radiographs showed that FTA was 174°, HKA was neutral, and the patella was located on the femoral component groove (Figure 3).

## 3. Discussion

To the best of our knowledge, this is the first case report to demonstrate the therapeutic potential of mobile bearing TKA with lateral retinaculum release for TF joint OA with permanent patellar dislocation. Mobile bearing TKA allows rotation between the polyethylene insert and the tibial component. It can theoretically adjust rotational malalignment by its self-align feature. Moreover, it has been reported that mobile bearing insert significantly improved patellar tracking with decreased patellofemoral contact stresses than fixed bearing insert in an intraoperative kinematic study [6]. This case demonstrated that some cases of permanent patellar dislocation with TF joint OA can be treated by mobile bearing TKA with lateral retinaculum release, without any other proximal or distal realignment surgical procedures.

Permanent patellar dislocation in adults is a relatively rare condition. Anatomical features of permanent patellar dislocation are as follows: valgus knee, trochlear groove abnormality, external tibial torsion, and hypoplasia of the patella [8]. There are currently several surgical options to correct the abnormal anatomy of patellar dislocation: proximal realignment (such as lateral retinaculum release) [9, 10], vastus medialis advancement [9, 10], medial transfer of the tibial tuberosity [9, 10], medial patellofemoral ligament (MPFL) reconstruction [11], trochleoplasty [1], and combinations of these surgical techniques.

Permanent patellar dislocation with TF joint OA is an even rarer condition. Neglected permanent patellar dislocation can cause valgus deformity and osteoarthritis in the lateral compartment [2]. These anatomical features of permanent patellar dislocation can be managed by TKA: vertical osteotomy to mechanical axis can correct valgus deformity, femoral component can correct trochlear groove dysplasia, mobile bearing insert can improve external tibial torsion, and patella replacement can correct patellar hypoplasia. Considering these advantages of TKA, it is reasonable to conclude that TKA can be utilized to treat permanent patellar dislocation. Implant positioning is also associated with patellar tracking during TKA. It is known that femoral and tibial components positioned in internal rotation cause patellar maltracking [12, 13], lateral positioning of the femoral component improves patellar tracking [14], and medial positioning of the patellar component results in smooth patellar tracking [15]. Furthermore, mobile bearing TKA can theoretically adjust rotational malalignment by its self-align feature and improve patellar tracking and patellofemoral contact stress [6].

Several papers have reported that medial transfer of the tibial tuberosity is useful for repositioning the dislocated patella during TKA [3, 4]. Other papers have reported that vastus medialis advancement with TKA is useful for this condition [5, 16]. Matsushita et al. reported successful clinical outcomes of TKA with MPFL reconstruction for TF joint OA with chronic patellar dislocation [17]. To our knowledge, however, there is no report about TF joint OA with permanent patellar dislocation treated by mobile bearing TKA with lateral retinaculum release. This case demonstrated that some cases of permanent patellar dislocation with TF joint OA can be treated by mobile bearing TKA with lateral retinaculum release, without any other proximal or distal realignment surgical procedures.

Based on these previous reports and our experience of this clinical case, the implant's precise position and alignment during TKA could settle anatomical abnormalities of permanent patellar dislocation, and the mobile bearing insert could contribute to stabilizing patellar tracking.
